# Unusual Regioselectivity in the Opening of Epoxides by Carboxylic Acid Enediolates

**DOI:** 10.3390/molecules13061303

**Published:** 2008-06-09

**Authors:** Luis R. Domingo, Salvador Gil, Margarita Parra, José Segura

**Affiliations:** Department of Organic Chemistry, Universitat de València, Dr. Moliner 50, 46100 Burjassot, Spain. Fax +34(9)63543831; E-mails: Salvador.gil@uv.es; jofranse@uv.es; Luis.Domingo@uv.es

**Keywords:** Lactones, lithium chloride, nucleophilic addition, regioselectivity, diastereoselectivity

## Abstract

Addition of carboxylic acid dianions appears to be a potential alternative to the use of aluminium enolates for nucleophilic ring opening of epoxides. These conditions require the use of a sub-stoichiometric amount of amine (10% mol) for dianion generation and the previous activation of the epoxide with LiCl. Yields are good, with high regioselectivity, but the use of styrene oxide led, unexpectedly, to a mixture resulting from the attack on both the primary and secondary carbon atoms. Generally, a low diastereoselectivity is seen on attack at the primary center, however only one diastereoisomer was obtained from attack to the secondary carbon of the styrene oxide.

## Introduction

Epoxides have been recognized among the most versatile compounds in organic synthesis, not only as final products [1] but as key intermediates for further manipulations. Accordingly, new synthetic developments are continuously being published [2]. Due to its high ring strain (around 27 Kcal/mol) its ring-opening, particularly with carbon-based nucleophiles, is a highly valuable synthetic strategy [3]. The usefulness of epoxides in S_N_2 reactions has been studied and important limitations precisely due to its key mechanistic aspect have been detected [2], namely, that the displacement of the leaving group is sensitive to both the substitution pattern of the involved carbon atom and the bulkiness of the nucleophile employed. Hence, a number of potentially useful reactions are kinetically hindered or even outright impossible. Two simple examples underline these restrictions: first, intermolecular epoxide openings by means of S_N_2 reaction usually do not occur at the higher substituted carbon atom and second, reactions of epoxides with sterically demanding substituents are frequently too slow to be preparative useful.

On the other hand, despite the venerable place held by enolates as carbon-based nucleophiles for organic synthesis, typical lithium enolates of esters and ketones do not react directly with epoxides [3]. Better results can generally only be attained by using the corresponding aluminium ester or lithium amide enolates. γ-Hydroxycarboxylic acids derivatives are rarely accessed via epoxide ring opening of lithium enolates [4] and some alternative routes have been proposed [5]. Our group has developed efficient conditions for the addition of lithium dianions of carboxylic acids to epoxides as an alternative route to aluminium enolate chemistry. The use of a sub-stoichiometric amount of amine for the dianion generation permits minimization of other nucleophiles present in the reaction medium, thus avoiding the generation of amino alcohols. This, along with an activation of the epoxide with LiCl, allows obtaining good yields for the additions to primary and secondary epoxides [6]. For primary epoxides, γ-lactones are directly obtained, thus avoiding a further cyclization step.

## Results and Discussion

With the aim of understanding the scope and limitations of this method, we discuss here its application to other enediolates and dienediolates derived from carboxylic acids, focusing on the regio and diastereoselectivity of this reaction.

**Scheme 1:**
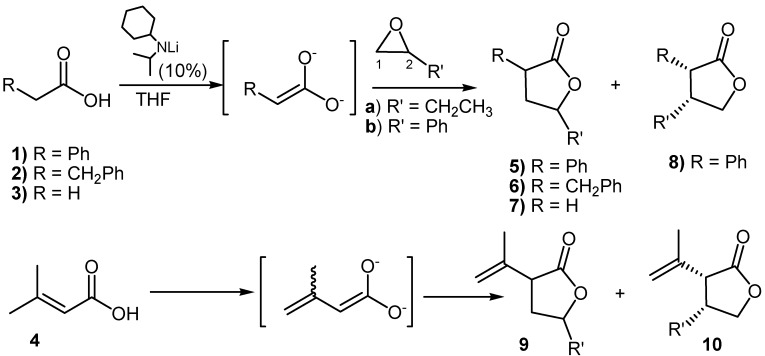
Additions of dienediolates of carboxylic acids to epoxides.

Here, we report the results obtained upon the addition of 1,2-epoxybutane and styrene oxide (**a** and **b**) to enediolates generated from acids **1-4** when a sub-stoichiometric amount (0.1 equiv.) of lithium cyclohexylisopropylamide is used to generate the dianion (Scheme 1). Standard conditions refer to 16 h reaction time at room temperature and inverse addition of LiCl (1 equiv.) as activator. Inverse addition involved the addition of the dianion solution to a mixture of the epoxide with LiCl in THF [7]. Any factors that might have affected the yield or stereoselectivity are studied in order to rationalize the results that are summarized in Table 1.

**Table 1:** Addition of carboxylic acid ene and dienediolates to epoxides.

Entry	Products	Yields	Regioselectivity	Diastereoselectivity^a^	Conditions^b^
			C-1/C-2 attack	*Anti/Syn*	
1	**5a**	93	100 : 0	52 : 48	
2	**6a**	48	100 : 0	66 : 34	
3	**9a**	77	100 : 0	53 : 47	
4	**9a**	70	100 : 0	56 : 44	0 ºC
5	**9a**	60	100 : 0	63 : 37	-40 ºC
6	**5b + 8b**	96	79 : 21	56 : 44	
7	**5b + 8b**	68	62 : 38	50 : 50	0 ºC
8	**5b + 8b**	86	70 : 30	63 : 37	without LiCl
9	**5b + 8b**	80	52 : 48	58 : 42	direct addition
10	**5b + 8b**	70	75 : 25	60 : 40	LiF
11	**5b + 8b**	72	84 : 16	63 : 37	LiBr
12	**6b**	50	100 : 0	63 : 37	
13	**7b**	48	100 : 0		
14	**9b + 10b**	46	84 : 16	51 : 49	0 ºC
15	**9b + 10b**	77	100 : 0	53 : 47	

^a^ Diastereoselectivity of products: **5**, **6**, **7** and **9**. Only one diastereoisomer is obtained for **8** and **10**; ^b^ Deviations from standard conditions

**Figure 1:**
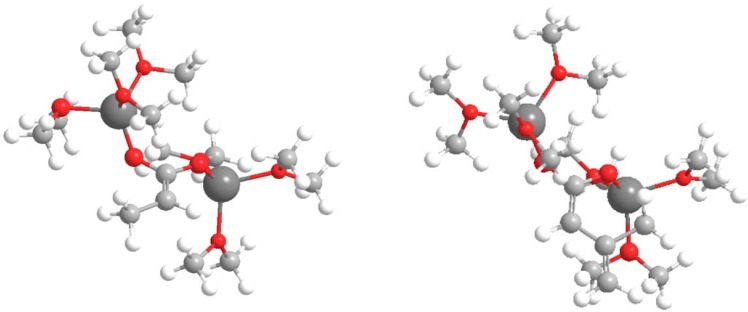
B3LYP/6-31G* optimized structures of the dienolates of propionic and dimethylacrylic acid (**4**).

It is well known that α,β-unsaturated carboxylic acid-derived dienediolates [eg. dimethylacrylic acid (**4**)] behave as ambident nucleophiles through either their α- or γ-carbon atoms, leading to single or predominant adducts when reacted with electrophiles under appropriate conditions [7]. Thus, α-attack is favoured for irreversible reactions whereas γ-adducts are obtained under equilibrium conditions. Nevertheless, the lithium dienediolates exist in solution as complex ion pair aggregates and the available data confirm the complexity present in these aggregated reactive species, whose reactivity and selectivity can be influenced by many different factors. Despite the longer reaction times, 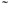 good α-regioselectivity is observed in this case following the trend of reactions with α-bromoacetonitrile [8]. This is consistent with the higher charge calculated for the α-position that slightly favours attack at this site, that can be quantitative with less reactive electrophiles. The Fukui 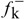 functions [9] for electrophilic attacks,, for the dienolates of propionic and dimethylacrylic acid (**4**) were calculated. Propionic acid was calculated as a model of non-conjugated acids **2** and **3**. Firstly, the structures of the corresponding dienolates were fulfil optimized using DFT methods at the B3LYP/6-31G* level [10, 11] with the Gaussian 03 suite of programs [12]. Previous theoretical structural studies of dienolates in solution have indicated the necessity to include the counter ions Li^+^ solvated with three solvent molecules (Et_2_O) [13]. The geometry of the corresponding optimized dianions is given in Figure 1.

These anions present an extended planar π system, the two lithium cations being coordinated to the two carboxyl oxygen atoms. Analysis of the Fukui functions for electrophilic attack at the dienolate of the propionic acid indicates that the α-carbon presents the largest value, 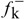 = 0.57, being this position the preferred for an electrophilic attack. For the dienolate of acid **4** analysis of the Fukui functions shows a charge delocalization along the conjugated π system. The corresponding values, 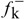 = 0.41 at the α-carbon and 0.31 at the γ-carbon, show a larger nucleophilic activation at the α-position than the γ-one. Therefore it will be expected the reaction to be regioselective towards reagents that favour a S_N_2 mechanism, being the major products those results from attacks to the α-carbon.

As expected [14], quantitative nucleophilic attack at the less hindered site of the aliphatic epoxide is always observed. With styrene oxide, attack at the primary site lead again to the major product, but a variable amount (16 to 48%) of products from attack at the more hindered position is obtained when dianions containing a π-extended moiety are used. These results contrast with the reactivity exhibited by styrene oxide with other nucleophiles where a regioselective attack the more susbtituted carbon atom occurs [14, 15]. This has been attributed to the fact that the unsaturated group facilitates, in the presence of Lewis acids, the polarization of the C-O bond and development of a positive charge at the benzylic carbon atom [15]. This electronic control does not operate for reactions with aluminium enolates as no products from attack to the most hindered position had been detected [3]. These results suggest that the nucleophilic attack to epoxides from enolates is sterically controlled. There is not a clear explanation where steric or electronic effects are critical to control the outcome of the different examples described.

The main difference between dianions derived from aliphatic and π-extended diolates is the higher charge localization in the former and thus, a S_N_2 like transition state is expected in their reaction with epoxides. This would lead to a major attack to the less substituted site irrespective of the substituent on the epoxide.

On the other hand, the lower nucleophility combined with less localized charges in π-extended dienediolates would lead to a less reactive behaviour. Thus, only epoxides with a certain degree of activation (by lithium complexation on the oxygen, for instance) will be attacked. In this case, the mixture observed with phenyl substituted epoxides may be due to the fact that steric effects would favor attack to the less substituted site whereas electronic effect would favour a major charge in the benzylic carbon atom. In the case of aliphatic epoxides, the electronic effect is likely to be less important.

We have studied the diastereoselectivity of this reaction. Although *trans* γ-lactones are always isolated as the major product, a low diastereoselectivity is observed for products **5**-**7** and **9** where attack to the less hindered position occurs. We have evaluated the influence of several factors, such as temperature, type of Lewis acid (LiF, LiBr, LiCl) or experimental set-up, on the diastereoselectivity but only slight changes were observed. LiCl play a double role: it can be acting as a disaggregating agent of the dienediolate and secondly as an activating agent in the opening of the epoxide througt its coordination to the oxygen atom. It is well known that different halides can be responsible to the stereoselectivity in enolate reactions [6, 7], but in this case its influence in the diastereoselectivity is low.

**Figure 2:**
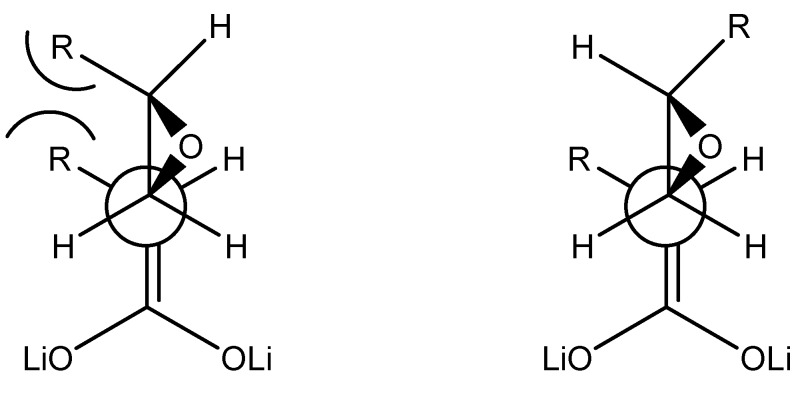
Possible approaches of dianions of carboxylic acids to the less substituted carbon in epoxides.

Considering the pre-transition state for S_N_2 type reaction described by Taylor [3] (Figure 2), the bulky groups appears to be too far from each other to show a significant effect. More significant diastereoselective results for other enolates in favour of the *cis* isomer are explained by steric hindrance of “R” groups [16]. In our case, the highly aggregated nature of enediolates might play a role as changes in the temperature, which are known to affect the aggregation order, promote changes in the diastereoisomeric ratio. A higher coordination on the lithium atom *anti* to the R group in the dianion is likely and may lead to a hindrance with the R group in the epoxide that overcomes that of both R groups.

**Figure 3:**
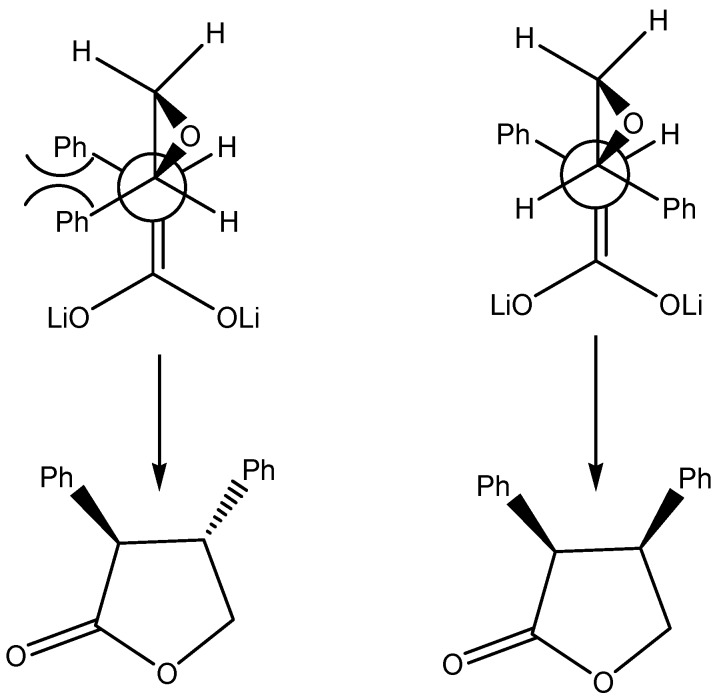
Possible approaches of dianions of carboxylic acids to the more substituted carbon in epoxides.

For products **8** and **10** only *cis*-diastereoisomers are observed. Using a similar approach as above to explain the pre-transition state, it can be seen that the one leading to the anti isomer would result in a destabilizing steric interaction (Figure 3) and, therefore, only *cis* lactones are obtained.

## Conclusions

We have developed a new approach to the synthesis of γ-lactones by addition of carboxylic acid enediolates to epoxides as an alternative to aluminium enolate chemistry. Study of the regio- and diastereoselectivity of this reaction indicates that is is highly regioselective to give 3,5-disubstituted lactones, except for reaction between styrene oxide and π-extended enolates, where variable amounts of the correponding 3,4-disubstituted ones are obtained. Diastereoselectivity is low for the 3,5-disubstituted lactones but only one diastereoisomer is obtained for 3,4-disubstituted ones.

## Experimental

### General

^1^H- and ^13^C-NMR spectra were recorded for CDCl_3_ solutions on a Bruker Avance 400 MHz spectrometer at 400 and 100 MHz, respectively. High resolution mass spectra were determined with a Fison VG Autospec spectrometer. Flash Column Silica Gel of 230-400 mesh (Scharlau) was used for flash column chromatography, with hexane/ethyl acetate mixtures as eluents. All reactions were carried out in oven dried glassware under argon atmospheres, using standard conditions for exclusion of moisture, in THF freshly distilled from blue benzophenone ketyl and with amines distilled from CaH_2_ and stored over molecular sieves and kept under Ar. The BuLi used was 1.6M in hexane. The solution’s exact concentration was periodically checked before use. The reaction temperature (-78^o^C) was achieved by cooling with a CO_2_/acetone bath and 0^o^C achieved by an ice/water bath. Organic extracts were dried over anhydrous MgSO_4_, and solutions were evaporated under reduced pressure with a rotatory evaporator and a bath at 40^o^C.

### General procedure for addition reactions.

Carboxylic acid (2.25 mmol) in THF (2 mL) was slowly added at -78 ºC to stirred lithium amide [for sub-stoichiometric conditions: cyclohexylisopropyl amine (0.45 mmol) and BuLi (4.5 mmol)] in THF (2 mL), according to the method already described. [6] The solution was stirred for 30 min at 0 ºC and cooled again to -78 ºC. The dianion mixture was added at -78 °C through the needle to a flask containing epoxide (2.25 mmol) and LiCl (94.4 mg, 2.25 mmol) in THF (2 mL) and then the solution was stirred for 16 hours at room temperature. The reaction was quenched with water (20 mL) and the mixture extracted with diethyl ether (3 x 15 mL). The aqueous layer was acidified under ice-bath cooling by careful addition of conc. hydrochloric acid, and then extracted with ethyl acetate (3 x 15 mL). The organic layer was washed with brine and dried (MgSO_4_). Evaporation of solvent gave the crude acid reaction mixture. For analytical purposes the products were isolated by column chromatography.

(3R^*^,5R^*^)-3,5-Diphenyl-3H-dihydro-2-furanone (**5b** major diastereoisomer): ^1^H-NMR: δ (ppm) = 7.34 – 7.26 (m, 10H, ArH), 5.62 (dd, J_1_=7.3, J_2_=5.4 Hz, 1H, CH-O), 3.88 (t, J=8.5 Hz, 1H, CHC=O), 2.78 (ddd, J_1_=13.0, J_2_=8.3, J_3_=7.6 Hz, 1H, CH_2_CH-O), 2.66 (ddd, J_1_=13.0, J_2_=9.0, J_3_=5.4 Hz, 1H, CH_2_CH-O); ^13^C-NMR: δ (ppm) = 177.0 (C=O), 139.4 (C-Ar), 136.5 (C-Ar), 129.1, 128.9, 128.4, 127.7, 125.1 (8CH-Ar), 78.8 (CH-O), 45.2 (CHC=O), 39.3 (CH_2_); MS: *m/z* (%) = 238 (M^+^, 3), 194 (M^+^-CO_2_, 100), 193 (M^+^-CO_2_H, 49), 117 (M^+^-CH_2_CHC_6_H_5_, 14), 116 (M^+^-HCH_2_CHC_6_H_5_, 28), 103 (M^+^-HOCOCHC_6_H_5_, 20), 77 (C_6_H_5_^+^, 25); HRMS: *m/z* calcd. for C_16_H_14_O_2_ [M^+^]: 238.0994 ; found: 238.0992.

(3R^*^,5S^*^)-3,5-Diphenyl-3H-dihydro-2-furanone (**5b**, minor diastereoisomer): ^1^H-NMR: δ (ppm) = 7.36 – 7.26 (m, 10H, ArH), 5.45 (dd, J_1_=10.8, J_2_=5.6 Hz, 1H, CH-O), 3.97 (dd, J_1_=12.8, J_2_=8.4 Hz, 1H, CHC=O), 3.00 (ddd, J_1_=12.8, J_2_=8.4, J_3_=5.6 Hz, 1H, CH_2_CH-O), 2.33 (ddd, J_1_=12.8, J_2_=12.8, J_3_=10.8 Hz, 1H, CH_2_CH-O); ^13^C-NMR: δ (ppm) = 176.4 (C=O), 138.7 (C-Ar), 136.1 (C-Ar), 129.1, 128.9, 128.7, 128.4, 128.1, 125.6 (8CH-Ar), 79.1 (CH-O), 47.6 (CHC=O), 40.5 (CH_2_); MS: *m/z* (%) = 238 (M^+^, 4), 194 (M^+^-CO_2_, 100), 193 (M^+^-CO_2_H, 52), 179 (46), 165 (12), 117 (M^+^-CH_2_CHC_6_H_5_, 27), 116 (M^+^-HCH_2_CHC_6_H_5_, 57), 115 (63), 104 (M^+^-OCOCHC_6_H_5_, 28), 103 (M^+^-HOCOCHC_6_H_5_, 41), 91 (C_7_H_7_^+^, 32), 77 (C_6_H_5_^+^, 47); HRMS: *m/z* calcd. for C_16_H_14_O_2_ [M^+^]: 238.0994 ; found: 238.0991.

(3R^*^,4S^*^)-3,4-Diphenyl-3H-dihydro-2-furanone (**8**): ^1^H-NMR: δ (ppm) = 7.08 (m, 6H, ArH), 6.80 (m, 4H, ArH), 4.68 (ddd, J_1_=9.4, J_2_=6.2, J_3_=4.5 Hz, 2H, CH_2_-O), 4.21 (d, J=8.5 Hz, 1H, CHC=O), 3.96 (dddd, J_1_=8.5, J_2_=6.0, J_3_=4.7 Hz, 1H, CHCH_2_-O); ^13^C-NMR: δ (ppm) = 176.2 (C=O), 136.7 (C-Ar), 133.0 (C-Ar), 129.4 (CH-Ar), 128.9 (CH-Ar), 128.8 (CH-Ar), 128.4 (CH-Ar), 128.3 (CH-Ar), 128.2 (CH-Ar), 127.9 (CH-Ar), 127.4 (CH-Ar), 127.3 (CH-Ar), 125.6 (CH-Ar), 71.3 (OCH_2_), 52.0 (CHC=O), 47.7 (CHCH_2_O); MS: *m/z* (%) = 238 (M^+^, 100), 194 (M^+^-CO_2_, 27), 193 (M^+^-CO_2_H, 30), 178 (M^+^-COOCH_2_, 26), 77 (C_6_H_5_^+^, 6); HRMS**:** *m/z* calcd. for C_16_H_14_O_2_ [M^+^]: 238.0994 ; found: 238.1008.
